# EU pharmacovigilance regulatory requirements of anticancer biosimilar monoclonal antibodies

**DOI:** 10.1007/s11096-018-0709-6

**Published:** 2018-08-09

**Authors:** Sara Francescon, Giulia Fornasier, Paolo Baldo

**Affiliations:** Pharmacy Unit, Directorate Department, Centro di Riferimento Oncologico CRO Aviano, National Cancer Institute, Aviano, Italy

**Keywords:** Adverse drug reaction, Biosimilars, Legislation, Monoclonal antibody, Oncology, Pharmacovigilance, Safety, Signal detection

## Abstract

An increasing number of innovative oncology monoclonal antibodies (mAbs) have been introduced into the global market, and biosimilar versions have now also been approved in Europe. Being complex to develop and difficult to manufacture, the biosimilar is a drug similar but not identical in physicochemical characteristics, efficacy, and safety to an original biological drug already approved in the European Union, for which marketing exclusivity rights have expired. Generally, the safety monitoring of biosimilars follows the same requirements that apply to all biologicals, even if specific pharmacovigilance measures exist and some of them are still being debated. The manufacturing process, immunogenicity, traceability, and extrapolation of indication are keywords which may impact on the achievement of additional knowledge about the safety of a biosimilar mAb. In this article, we aim to discuss elements that play a central role in the pharmacovigilance legislation of biosimilar mAbs.

## Introduction

Biological products are complex structures because of their basic protein structure and other modifications that they undergo during their maturation, generating a complex mix of the same protein under various isoforms (referred sometimes as *intra* changes). According to the Directive 2001/83/EC [1], a biological medicinal product is a product that contains an active substance that is produced by or extracted from a biological source. The active substance could derive from blood, plasma, recombinant DNA technology, etc.

A biosimilar is a version of a biological medicinal product (called the *originator* or *reference* dru*g*) already authorized in the European Union with demonstrated similarity in physicochemical characteristics, efficacy, and safety, based on a comprehensive comparability exercise [2].

In the oncology setting, Europe is essentially concerned by biosimilars of Erythropoiesis Stimulating Agents (ESAs) (e.g., epoetin alfa) and Colony Stimulating Factors (CSFs) (e.g., filgrastim) and recently, the market is affected by the arrival of biosimilars of monoclonal antibodies (mAbs) (see Table 1).

**Table 1:** List of current EU-authorised biosimilars in the field of anticancer therapy, up to November 2017

Name	Active substance	Type of treatment	Date of marketing authorisation	Clinical indication
Abseamed	Epoetin alfa	Supportive care	28/08/2007	Anemia Cancer Kidney Failure, Chronic
Binocrit	Epoetin alfa	Supportive care	28/08/2007	Anemia Kidney Failure, Chronic
Epoetin alfa hexal	Epoetin alfa	Supportive care	28/08/2007	Anemia Cancer Kidney Failure, Chronic
Retacrit	Epoetin zeta	Supportive care	18/12/2007	Anemia Blood Transfusion, Autologous Cancer Kidney Failure, Chronic
Silapo	Epoetin zeta	Supportive care	18/12/2007	Anemia Blood Transfusion, Autologous Cancer Kidney Failure, Chronic
Accofil	Filgrastim	Supportive care	18/09/2014	Neutropenia
Filgrastim hexal	Filgrastim	Supportive care	06/02/2009	Cancer hematopoietic Stem Cell Transplantation Neutropenia
Grastofil	Filgrastim	Supportive care	18/10/2013	Neutropenia
Nivestim	Filgrastim	Supportive care	08/06/2010	Cancer hematopoietic Stem Cell Transplantation Neutropenia
Ratiograstim	Filgrastim	Supportive care	15/09/2008	Cancer hematopoietic Stem Cell Transplantation Neutropenia
Zarzio	Filgrastim	Supportive care	06/02/2009	Cancer hematopoietic Stem Cell Transplantation Neutropenia
Blitzima	Rituximab	Active treatment	13/07/2017	Leukemia, Lymphocytic Chronic, B-Cell Lymphoma, Non-Hodgkin
Ritemvia	Rituximab	Active treatment	13/07/2017	Lymphoma, Non-Hodgkin Microscopic Polyangiitis Wegener Granulomatosis
Rituzena	Rituximab	Active treatment	13/07/2017	Leukemia, Lymphocytic, Chronic, B-Cell Lymphoma, Non-Hodgkin Microscopic Polyangiitis Wegener Granulomatosis
Rixathon	Rituximab	Active treatment	15/06/2017	Arthritis, Rheumatoid Leukemia, Lymphocytic, Chronic, B-Cell Lymphoma, Non-Hodgkin Microscopic Polyangiitis Wegener Granulomatosis
Riximyo	Rituximab	Active treatment	15/06/2017	Arthritis, Rheumatoid Leukemia, Lymphocytic, Chronic, B-Cell Lymphoma, Non-Hodgkin Microscopic Polyangiitis Wegener Granulomatosis
Truxima	Rituximab	Active treatment	17/02/2017	Arthritis, Rheumatoid Leukemia, Lymphocytic, Chronic, B-Cell Lymphoma, Non-Hodgkin Microscopic Polyangiitis Wegener Granulomatosis

Unlike ESAs and CSFs, mAbs are complex proteins with high molecular weight; they are produced by clones and they are used in research, diagnosis and to treat life-threatening diseases such as cancer.

Because of their high specificity, high affinity to targets and fewer side effects, mAbs have revolutionized the clinical practice [3]. However, the complexity of these molecules has an impact on aspects which are essential to define a medicinal product: quality, efficacy, and safety.

Through this commentary, we would highlight some features that play a central role in pharmacovigilance legislation of anticancer biosimilar mAbs, and therefore, on their safety profile.

## The legislative framework and pharmacovigilance

All biosimilars are subject to a centralized European approval process. A biosimilar’s applicant for a Marketing Authorization (MA) must follow all requirements contained in the Directive 2001/83/EC [1], implemented by the European Medicines Agency (EMA) Committee for Medicinal Products for Human Use and EMA specific guidelines [2].

The biosimilar’s MA is based on the demonstration of biosimilarity with its *originator* in terms of quality, efficacy, and safety in accordance with the comprehensive comparability exercise.

The comparability exercise is a step-wise process which consists of a series of head-to-head comparability studies of the biosimilar with its *originator*. The first step is based on comparative quality studies (physicochemical and biological studies). These studies, essential for abridging the non-clinical and clinical studies, are the most sensitive to detect differences between the biosimilar and its *originator*. Non-clinical studies (the second step) are pharmacodynamic and toxicology studies. The backbone of the second step is characterized essentially by *in vitro* studies. The last step are the comparative clinical studies, whose purpose is to confirm biosimilarity and to exclude any clinically relevant difference in terms of efficacy and safety.

Generally, non-clinical studies are always required by regulators and their extent is tailored to biosimilar’s characteristics. Clinical studies might be waved where such studies may be considered unnecessary. However, as expected for complex molecules as mAbs, non-clinical and clinical studies should always be conducted.

Regarding safety, pharmacovigilance is fundamental to obtain additional data on the safety profile of an anticancer biosimilar mAb in the real-world setting.

All biosimilar medicines follow the same requirements that apply to all biological medicines, based primarily on Good Pharmacovigilance Practices (GVP) [4]. The Marketing Authorisation Holder (MAH)/applicant must have an appropriate risk management system and must submit a Risk Management Plan (RMP) which is described in the GVP Module V [5]. The RMP is tailored to each product and its updated is assessed on case-by-case basis. If no impact on the safety and efficacy profile is expected, there is no need to update the RMP. The RMP aim is to ensure that the drug’s benefits exceed the risks by the greatest achievable margin and it includes actions intended to reduce, prevent or minimise risks.

The RMP part III is particularly important; it consists of the Pharmacovigilance plan to identify whether, and which, routine or additional pharmacovigilance activities are needed.

Routine pharmacovigilance is the primary minimum set of activities required for all medicinal products accordingly to obligations set out by the Regulation (EC) No 726/2004 [6] and the Directive 2001/83/EC.

The most important activities of routine pharmacovigilance are Adverse Drug Reactions (ADRs) reporting and signal detection. As part of routine pharmacovigilance, MAHs/applicants must collect all reports of suspected ADRs and submit Periodic Safety Update Reports (PSURs) to regulators.

When necessary, the EMA or a national competent authority may impose on the MAH/applicant additional pharmacovigilance activities. These are non-routine measures or activities performed to provide additional information about the long-term safety profile of a medicinal product or to investigate a safety concern. Examples of additional pharmacovigilance activities are the Post-Authorisation Safety Studies (PASS), which may be clinical, non-clinical, or observational studies normally conducted after approval of the medicinal product. Normally, if a PASS has been requested for the *reference* mAb, it could be also requested for the biosimilar mAb. Other examples of additional PV activities are the use of patients registries or additional long-term immunogenicity data.

Finally, biosimilars, as well as all biological medicines approved after the 1st January 2011, are subject to additional monitoring and they are included in the ‘List of Drugs Subject to Additional Monitoring’. This means that these medicines are being monitored particularly closely by regulatory authorities after their approval or during a particular time of their life-cycle. A black triangle symbol displayed in the Summary of Product Characteristics (SPCs) identifies all medicinal products inserted in the list. When further studies confirm that the product has a favourable risk-benefit balance, the EMA’s Pharmacovigilance Risk Assessment Committee (PRAC) removes it from the list.

## Manufacturing process

Unlike chemically synthesized medicines, biological medicinal products (biosimilars included) are complex structures and manufacturing changes happen frequently both for originators and for biosimilars. A change can be defined as:

- *Intra,* a pre-change product versus a post- change product (for example among different batches of the same product). *Intra* changes are related to the choice of cell line, fermentation, purification, and the formulation and they can determinate some differences between different batches of the final product.
- *Inter,* a manufacturer A-product versus a manufacturer B-product. *Inter* changes are based on the fact that the development of a biosimilar requires the establishment of a new manufacturing process because there is usually no direct access to originator companies’ proprietary data.

The biosimilar company has to characterize as much as possible *the originator*, then it has to do a sort of ‘reverse engineering’, and finally to manufacture a product which is as closest as possible with *the originator*.

In the field of biological medicinal products, it is commonly said that ‘the process makes the product’since the biosimilar is defined by its manufacturing process specific to the active substance and to the finished product (as for *the originator*). The complex manufacturing process and *intra*/*inter* changes can affect the drug’s safety, quality and efficacy profile. Therefore, the EMA obliges the MAH/applicant to test the pre-version and the post-version of the product to demonstrate that both efficacy and safety remain the same, before and after the change.

## Immunogenicity

Immunogenicity is an unexpected and unwanted immune response that may impact on the risk-benefit balance of a medicinal product, and this has a particular relevance for biological medicines and biosimilars. Its potential clinical consequences could result from a transient appearance of antibodies to loss of drug’s efficacy or serious adverse reactions.

The risk of immunogenicity is assessed for all biological and not specific for biosimilars, and it has to be evaluated during the entire biosimilar’s life-cycle in relation to the totality of the evidence, from comprehensive physicochemical/biological assays and non-clinical studies to the post-marketing setting (through additional pharmacovigilance activities).

In fact, as immunogenicity could depend on several factors related to the manufacturing process, patient’s characteristics, disease, and treatment, data available at the time of approval for a biosimilar may not be sufficient to robustly assess the safety and its correlation to clinical outcomes. Therefore, some adverse reactions and risks (immunogenicity included) will only be discovered in the post-authorisation phase, improving the detection of new signals of harm for patients. For this reason, the risk of immunogenicity of a biosimilar is assessed in relation to the totality of evidence.

## Extrapolation of indication

Extrapolation of indications is a well-accepted regulatory and scientific principle of biological development, and an extensive experience has been gained with biosimilars of ESAs and CSFs. A lot of experience on extrapolation of indication has also been obtained for mAbs in other indication.

The efficacy and safety data extrapolation to other indications of the reference mAb, not studied during development, is possible when the similarity with the *originator* has been convincingly demonstrated and scientifically justified in a key indication. This means that key features of the biosimilar (e.g. structure, function, pharmacology, mechanism of action, study population, and clinical setting) have shown a ‘high similarity’ with those of the reference product, and so efficacy and safety data can be expected to be the same.

Sometimes all these features are not always well-known or completely defined for anticancer biosimilar mAbs. As reported by Cortés et al. [7], it is not ever possible to define the precise mechanism of action of a medicinal product since the mAb may act with several mechanisms that result in its efficacy or safety. Furthermore, the efficacy and safety similarity may be influenced by factors not directly attributable to differences between reference mAb and biosimilar mAb. These factors could be the setting (neoadjuvant/adjuvant, metastatic), the disease heterogeneity, co-morbidities, concomitant medications and interference by ongoing anticancer treatment. For example, the absence of a treatment free-phase during treatment for metastatic disease allow to hypothesize that this setting would be less sensitive and accurate to assess the efficacy and safety similarity between the biosimilar mAb and its *reference*.

Therefore, the concept of extrapolation of indications in the context of biosimilar mAbs is not automatic and it requires always a case-by-case evaluation, based on clinical experience gained on the *originator*, on the available scientific literature, and on the totality of data obtained through the comparability exercise.

## Traceability

Traceability is essential to better know ADRs history and chronology. The risks due to a heterogeneous and undefined European policy regarding biosimilars’ switching, make traceability of fundamental importance.

Klein et al. [8] report that there is a possible relationship between the availability of the brand name and batch number information in ADRs reports for biological medicines. In fact, brand names are not routinely recorded in the clinical practice and moreover, batch numbers are poorly recorded overall [9].

Furthermore, the current terminology for ADRs reporting has no appropriate MedDRA coding [10] for ADRs resulting from switching to different biosimilars, so it would be necessary to introduce an appropriate MedDRA coding for this kind of situations.

Also, the naming can have important implications for traceability and safety monitoring for biosimilar mAbs. The most appropriate naming convention for biologics has been an area of significant debate. The international non-proprietary name (INN) fills in the need of a scientific nomenclature of molecules, that identifies them in an unequivocal and neutral way on an international level. When the molecule is not protected by a patent, the INN may be used by all producers competing on the market, associated with their own brand name. However, bearing the same INN does not mean the same thing in the field of biologicals. The EMA has not expressed any position concerning biosimilars’ naming: the biosimilar’s producer may use or not the same INN as the *originator*. Some producers use the same INN, others a different INN (e.g. *epoetin zeta*) and all these options are not in contradiction with the European regulation.

As many reports described INN, is not always possible to distinguish between ADRs reported for the biosimilar and ADRs reported for the *originator*, and this could be a problem for communications with Pharmacovigilance network. The unique identification of a biosimilar has a paramount importance to ensure the traceability of the medication in order to avoid compromising signal detection in pharmacovigilance [11].

## Conclusions

As discussed above, EU regulatory authorities have an extensive experience on biological medicinal products and guidelines are scientifically well-established.

Due to the intrinsic and extrinsic complexity of biosimilar mAbs, the compliance with the specific EU regulatory requirements certify the quality, efficacy, and safety of these medicines.

A deep knowledge of all these aspects is very important for commissions dealing with hospital formularies, for acquiring a comprehensive information and appropriately conducting the inclusion process of biosimilar mAbs into clinical practice.

## References

[1] Directive 2001/83/EC of the European Parliament and of the Council of 6 November 2001 on the Community code relating to medicinal products for human use [Internet]. Official Journal L 311, 28/11/2001 P. 0067–0128; [cited 2018 Apr 5]. http://eur-lex.europa.eu/legal-content/EN/TXT/HTML/?uri=CELEX:32001L0083&from=it.

[2] European Medicines Agency. Guideline on similar biological medicinal products (CHMP/437/04 Rev 1) [Internet]. [cited 2017 Dec 29]. http://www.ema.europa.eu/docs/en_GB/document_library/Scientific_guideline/2014/10/WC500176768.pdf.

[3] Alkan SS (2004). Monoclonal antibodies: the story of a discovery that revolutionized science and medicine. Nat Rev Immunol.

[4] European Medicines Agency—Pharmacovigilance—Good pharmacovigilance practices [Internet]. [cited 2017 Dec 31]. http://www.ema.europa.eu/ema/index.jsp?curl=pages/regulation/document_listing/document_listing_000345.jsp.

[5] European Medicines Agency. Guideline on good pharmacovigilance practices: module V—Risk management systems (Rev. 2) [Internet]. 2017 [cited 2018 Apr 5]. http://www.ema.europa.eu/ema/index.jsp?curl=pages/regulation/document_listing/document_listing_000345.jsp&mid=WC0b01ac058058f32c.

[6] Regulation (EC) No 726/2004 of the European Parliament and of the Council of 31 March 2004 laying down Community procedures for the authorisation and supervision of medicinal products for human and veterinary use and establishing a European Medicines Agency (Text with EEA relevance) [Internet]. OJ L, 32004R0726 Apr 30, 2004. http://data.europa.eu/eli/reg/2004/726/oj/eng.

[7] Cortés J, Curigliano G, Diéras V (2014). Expert perspectives on biosimilar monoclonal antibodies in breast cancer. Breast Cancer Res Treat.

[8] Klein K, Scholl JHG, Vermeer NS, Broekmans AW, Van Puijenbroek EP, De Bruin ML (2016). Traceability of biologics in The Netherlands: an analysis of information-recording systems in clinical practice and spontaneous ADR reports. Drug Saf.

[9] Vermeer NS, Straus SMJM, Mantel-Teeuwisse AK, Domergue F, Egberts TCG, Leufkens HGM (2013). Traceability of biopharmaceuticals in spontaneous reporting systems: a cross-sectional study in the FDA Adverse Event Reporting System (FAERS) and EudraVigilance databases. Drug Saf.

[10] MedDRA: ICH [Internet]. [cited 2018 May 14]. http://www.ich.org/products/meddra.html.

[11] Vermeer NS, Spierings I, Mantel-Teeuwisse AK, Straus SMJM, Giezen TJ, Leufkens HGM (2015). Traceability of biologicals: present challenges in pharmacovigilance. Expert Opin Drug Saf.

